# Correction: Who presents the greatest challenge in intellectual disability research- participants or health and research professionals?

**DOI:** 10.1371/journal.pone.0343139

**Published:** 2026-02-17

**Authors:** Rosemary Kelly, Laurence Taggart, Vivien Coates, Maria Truesdale, Alison Dunkley, Michelle Hadjiconstantinou, Kamlesh Khunti, Nicola Mills

Gary McDermott is not included in the author byline. Gary McDermott should be listed as the ninth author and affiliated with Research Fellow, School of Nursing and Midwifery, Queen’s University Belfast, United Kingdom. The contributions of this author are as follows: Developed/designed methodology, and wrote/reviewed & edited the paper.
