# Supplementary material for: Who presents the greatest challenge in intellectual disability research- participants or health and research professionals?
Source: PLoS One. 2025 Nov 3;20(11):e0332744. doi: 10.1371/journal.pone.0332744 (PMC12582445; doi:10.1371/journal.pone.0332744)
Supplement: S2 File — (PDF) [file pone.0332744.s002.pdf]

Supporting Information S2 File

Topic Guide for 1:1 interviews with professionals

Study Title: My Diabetes and Me

Exploring the barriers and enablers of identifying and approaching adults with learning disabilities (LD) for the My Diabetes and Me Study.

Opening statement:

Thank you for taking the time for this interview today. My name is [Interviewer's name] and I'm part of the My Diabetes and Me research team and I'm exploring how staff might identify and approach adults with LD for the My Diabetes and Me Study.

I want to remind you that there are no wrong answers and the interview can be stopped at any time.

WARM UP

Recording for the My Diabetes and Me study by [Interviewer's name] with [Staff ID] on [date]. Are you happy for this discussion to be audio-recorded?

□ Confirm the participant has given written consent and wishes to proceed with the interview

□ Remind the participant of the aim of the study and clarify the terms of confidentiality regarding this study as follows:

The aim of this research is to investigate the most effective means of identifying and approaching adults with LD and T2D for the My Diabetes and Me Study.

Your name will not be used in this study. Instead you will be allocated an ID number so your data remains anonymous. You are asked not to disclose specific patient cases but if you do the data will be anonymised.

□ Explain that the interview will take approximately 30 minutes.

INTERVIEWEE BACKGROUND

1. Can you briefly describe your clinical role with adults with LD?

   Prompt: Do you meet adults with LD who also have T2D? Are they usually accompanied by a carer/relative/friend?

   Are there any benefits/challenges of communicating through another person?

2. What is your understanding of and experience with RCTs?

   Prompt: Do you know what an RCT is? Have you ever been involved in one?

STRUCTURED EDUCATION

3. Do you think it is realistic for people with LD and T2D to take an active role in their self-management?

4. Are you aware of the structured education programme called DESMOND?

   Prompt: Based on the Information booklet shared with you prior to this interview

5. I'm interested in your general thoughts as to whether the DESMOND programme can be modified and used by those with LD.

   Prompt: If you are not familiar with DESMOND, do you feel structured education programmes can be designed/helpful for those with LD?

   PROVIDE INFORMATION ON STUDY DESIGN AND AIMS IF NECESSARY

6. Are you familiar with the modified version of this structured education programme – DESMOND-ID?

7. What do you think about this future study regarding DESMOND-ID?

SCREENING

8. Let's think a bit about the eligibility criteria and what you think about applying them in practice

   Prompt: Eligibility criteria:

   Adults with LD with T2D living in the community Aged ≥ 18 years

   Mild/moderate LD as confirmed by professional/medical records Willing to engage in a structured education programme

Ability to give informed consent

A carer/partner/advocate to accompany them if appropriate

Prompt: What do you think about these criteria?

Is there anything that would make you uneasy or uncomfortable about approaching people in that list?

Is information about the degree of LD likely to be well recorded and accessible? Is there anything missing?

9. Who would be responsible for determining study eligibility?

   Prompt: How might families/carers' opinions influence participant recruitment?

10. What do you think the main difficulties recruiting to this study will be?

    Prompt; What could we do to address those difficulties?

11. Can you tell me about the ways you have been prepared to screen people for this study?

    Prompt: Do you feel prepared to take on this role?

12. How would you go about identifying people who would be eligible to take part in this study?

    Prompt: How easy do you think this will be?

    What would you need in terms of time/resources to be able to do this?

INTRODUCING THE STUDY

13. Will you have any role in introducing/explaining the study to potential participants?

    Prompt: If YES, how would you plan to do that?

    How comfortable do you feel/think you might feel explaining the study and related concepts

    e.g. randomisation and rationale for study?

    What could we do to address any difficulties around this? If NO, who is it likely to be?

    Prompt: what do you think the study pathway/process for screening potential participants will look like in reality (how will the screening be done; who will assess eligibility and when; who/when/how will potential participants be approached; who/when/how will the study be discussed with potential participants?

QUESTIONS FOR THOSE ALREADY INVOLVED IN RECRUITMENT TO THE STUDY

[If screening logs are being completed and uploaded, check these prior to interviews. Note any patterns and raise for discussion e.g. large numbers screened but low numbers eligible or consenting].

14. Roughly how many people have you screened to find those that meet the inclusion criteria for the DESMOND-ID trial?

Prompt: You might want to refer to your screening log for this.

What do you think about the initial screening process to find those who may be eligible? (Straight forward/Difficult/Time consuming)

15. Approximately how many potential participants would you say you have identified so far, if any?

Prompt: You might want to refer to your screening log for this.

16. What would help with identifying potential study participants?

Prompt: Are different approaches needed for people with different degrees of disability? What might those be?

Would you feel comfortable considering those when screening people for inclusion?

17. Would there be anything we could do to help jog your mind about recruiting to this study?

Prompt: Are there any forms or alerts or reminders that might help?

18. Thinking about what previous colleagues said about introducing the study to potential participants, and with the last person you screened in mind, can you talk me through the process you used?

Prompt: How did you screen them; how did you determine eligibility; how did you approach the study with them (PIS and verbal); did they understand the concept of randomisation; what was their response; what was the final outcome?

Prompt: If they declined, do you have any sense of why they declined? Do you ever get potential participants asking you what you think is best? How do you respond?

19. How comfortable do you feel asking people to consider taking part?

Prompt: Are there particular types of people you feel more uncomfortable approaching and discussing the study with?

Why is that?

What do you tend to do in those situations?

Do you have any sense of what your colleagues do?

What is the discussion like when a carer/family member/friend is present too?

Was the accompanying person influential in the person with LD andT2D deciding to hear more about the study?

20. How much information do you give on the study at this point? How comfortable are you in exploring their reasons for their views etc?

Prompt: What study information do you have to hand (PIS; Information booklet; anything else you found helpful?)

21. What happens if someone is initially unsure about taking part - how did you respond?

Prompt: Did you try to encourage them to take part or did you remain neutral?

22. Finally, can you reflect on your experience of recruiting to date and what the main issues were for you.

Prompt: Specific challenges you weren't expecting

What caught you unawares?

Information you needed that you felt you didn't have

TO FINISH

We really hope you are able to continue helping us to recruit. Please remember the importance of recording your recruitment conversations so we can capture and share good recruitment discussions and address any challenges around the recruitment as we get into the main research study.

Do you have any final thoughts or suggestions that might increase recruitment? Thank you again for your time and your help with this study.

END
