# Supplementary material for: Who presents the greatest challenge in intellectual disability research- participants or health and research professionals?
Source: PLoS One. 2025 Nov 3;20(11):e0332744. doi: 10.1371/journal.pone.0332744 (PMC12582445; doi:10.1371/journal.pone.0332744)
Supplement: S3 File — (PDF) [file pone.0332744.s003.pdf]

My Diabetes & Me

Interview Topic Guide for Participants (Example)

Notes: The detailed version of the topic guide will be developed by the research team as part of the study. This topic guide is a flexible tool and may be revised as new areas of interest arise during the process of data collection. The wording of the question is for guidance only and can be varied to suit the natural style of the interviewer and the level of understanding of the participant. This topic guide will be adapted to use for those who are involved in this study.

The interview will confirm that:

- The purpose of the interview is to find out the participants' knowledge, views, experiences related to:
  - The My Diabetes and Me self-management programme
  - We want to find out whether a programme such as My Diabetes and Me can be implemented into the real world.
- The study-participant is not being tested. There are no right or wrong answers.
- The study-participants free to say as much or as little as they want.
- The content of the interview will not be divulged to their health care providers.

Recording to be started at this point if not already on.

To aid memory, visuals of the sessions/resources will be shown in the interview.

Possible areas of discussion

Venue / Group dynamic /Attendance

1. Was the venue convenient for you?

2. Was it easy to attend?

3. Was there any reason you were not able to attend?

4. How comfortable did you feel in the group?

The Education Programme

5. What did you think of the education?
   a. How did you find the format / delivery? (i.e. was straightforward, or easy to understand)

6. Was MD&M a good way to give you advice/help you needed? Why?
   Prompts:
   - What would you have preferred?
   - What, if anything, would you change about the MD&M sessions for keeping active, having a good diet?

- If a friend was thinking of maintaining a healthy lifestyle, how would you advise them to go about this?

7. How did you feel about the length/timing of the course? (Prompt: too short, too long)

8. How did you find the information on:
   - Diet
   - Physical Activity

9. Did you like having (name of carer/family member) with you in the programme? Elaborate.

10. Can you tell us if you felt the sessions helped in any way to change your eating, exercise? Prompt if yes, no, why).

11. Did you find setting a goal helpful? Tell me a bit about that?

12. Do you think other people should have this programme to help them with their diabetes, or not?

13. Was there anything you didn't/did like in the sessions?

14. How did you find the additional (booster) sessions that you attended

Research

1. Are you comfortable taking part in research?

2. Do you feel there are benefits in taking part in research? (elaborate)

3. What did you like/did not like about taking part in this research?

Closing Question

1. Is there anything that we didn't cover, that you would like to mention/comment?

Thank you for your time
