# Supplementary material for: Who presents the greatest challenge in intellectual disability research- participants or health and research professionals?
Source: PLoS One. 2025 Nov 3;20(11):e0332744. doi: 10.1371/journal.pone.0332744 (PMC12582445; doi:10.1371/journal.pone.0332744)
Supplement: S4 File — (PDF) [file pone.0332744.s004.pdf]

Supporting Information S4 File

Study Title: My Diabetes and Me

Code book example

Coding by RK
Gatekeeper interviews

Descriptor	Content	Action
**Already covered or within research team remit**		
Defining disability	GP teams can get to know clients in residential care well	Engage with GP practices re recruitment in these facilities
	Thinking outside the box to communicate with people with ID – one size doesn't not fit all	Recruiters to identify preferred method of communication with potential participant/ carer prior to recruitment interview
Family/informal carers	• Ensuring families understand the purpose of the study to get 'buy in'<br>• Families will only engage if they perceive the client to have a problem with their diabetes – will see no benefit to taking part otherwise<br>• Families/ potential participants may want local study centre	• Provide a one-page PIS for families/carers as well as potential participants<br>• Importance of PIS and carer's session [of DESMOND]<br>• Recruiters to identify nearest centre for participants to attend and discuss transport issues
GK: Clinical trials: knowledge or experience of	• Clarity needed around research processes and time frames<br>• Potential participants may be put off by the word 'trial'. Use 'study' throughout<br>• Several HCP pathways so information may be in different systems<br>• Helping staff at each stage to understand their role in the study	• Amend Protocol prior to main study to reflect learning from Internal Pilot<br>• Ensure study paperwork is clear about responsibilities<br>• Establish pathways through Internal Pilot<br>• Regular planning meetings to address any issues including training, what to tell potential clients, familiarisation with documents, RCT and DESMOND ID information pack

Descriptor	Content	Action
	• Role of senior staff in supporting screeners/recruiters<br>• Clear explanations about the two arms of the study<br>• Engage with GP practices to encourage 'buy in'<br>• Review outcomes to encourage uptake of main study	• Add updates to DESMOND newsletter circulated to practices and ?DOH website<br>• Cost-benefit analysis
GK: recruiting: systems and processes	• Use of Health Facilitators to encourage 'buy in'<br>• Data protection issues around handing over a large list of potential participants without their consent<br>• Managing amount of information provided to potential participants initially<br>• Supporting GP practices to endorse study<br>• Identify other pathways of potential participants	• Where available, to help engage GP practices<br>• Gatekeepers to provide anonymised screening lists until consent obtained<br>• Provision of one-page PIS to be sent out with invitation letter ahead of 12 page PIS<br>• Invitation letter to be sent on GP practice headed paper with researcher contact information or with signed GP slip to indicate GP's support for study<br>• Information packs to be provided to practices for mail merge<br>• Hold planning meetings with residential/day care staff/voluntary groups
Participants/clients	• Empowerment and equity<br>• Motivation	• Promote study as a self-management strategy<br>• Ensure equipoise during recruitment process<br>• Stress importance of attending all study sessions<br>• Use appropriate type and length of study information<br>• Provide positive feedback on individual's participation to make people feel valued<br>• Ensure on-going support to maintain motivation<br>• Careful explanation of benefits of both randomised groups

Descriptor	Content	Action
		• Emphasise benefits of group education and socialisation<br>• Make adequate time available during study sessions to ensure participant understanding
ID staff: residential care homes/community centres/adult centres	Promoting healthy lifestyle	Ensuring staff are aware of importance of diabetes management
**Possible actions to be taken**		
Defining disability	Transferring information between services	• Capturing information on potential participants kept across different pathway systems<br>• Explicit coding within GP practices to identify potential participants
Family/informal carers	Families may anticipate HCP support if they are unable to travel	Options for support with travel for participants if family members/support workers are unable to bring them to study sessions
GK: Clinical trials: knowledge or experience of	• Reimbursement for work related to study activity<br>• Research may not be a priority during current climate<br>• Concerns about participants being able to manage 7-week course	• Reimbursement fees payable during data collection<br>• Planning meetings to support/educate clinical teams<br>• Motivation to be discussed with potential participants at recruitment interview
GK: recruiting: systems and processes	• Generating list of potential participants	• Coding system within GP practices can facilitate generating lists if complied correctly
	• Identifying staff to talk to potential participants about the study	• Discussions with Community LD teams and primary care staff about clients they have regular contact with<br>• GP Practice staff who undertake LD reviews may use opportunity for study discussion

Descriptor	Content	Action
Participants/clients	Self-management/empowerment	Provide small financial incentive for time spent on the study
ID staff: residential care homes/community centres/adult centres	Capacity to help	• Hold discussions with mental health teams and community support workers to offer support<br>• Discuss with senior staff in residential care/day centres releasing staff to attend sessions with participants<br>• Discuss with senior staff in supported living facilities the importance of prioritising attendance at the study sessions
**Issues not yet decided on**		
GK: Clinical trials: knowledge or experience of	Researchers being directed only to those staff are confident will participate/benefit [Equipoise]	

Coding by VC

Themes (from interviews/recruitment discussions with client participants)

Diabetes Awareness

Participants aware they have Type 2 Diabetes
Differing awareness of symptoms and risks
Understanding of importance of diet and exercise

Encouraging participation

PIS read to most participants by carer
Not everyone remembers discussion about study including multiple visits
Info about vouchers remembered by all

Varying recall of info about QRI study
Having pictures and easy read format in PIS was helpful
Understood the study related to diabetes
Amount of information provided was daunting for some
Information needs broken down into single ideas for some participants
Checking understanding with specific questions at regular intervals important
Reading out items on consent form before signature further confirms understanding
Most participants understood process of randomization
Limited literacy skills
Reasonable recall of baseline measures performed
Mixed response to feeling able to ask questions
Care needed with terminology and tempo of speech
Understood most elements of study

Emotional labour for inclusivity

Most participants happy with baseline home visit but choice important
Understanding of attending group sessions
Mix of accompanied and unaccompanied attendance at group sessions
No concerns about being in group despite some participants experiencing social anxiety
Reassurance needed around transport
All happy to attend a clinic for future study appointments but would need to be accompanied
