# Supplementary material for: Who presents the greatest challenge in intellectual disability research- participants or health and research professionals?
Source: PLoS One. 2025 Nov 3;20(11):e0332744. doi: 10.1371/journal.pone.0332744 (PMC12582445; doi:10.1371/journal.pone.0332744)
Supplement: Attachment — Submitted filename: PlosOne_Peer review.pdf [file pone.0332744.s007.pdf]

Peer-Review PolsOne :

Who presents the greatest challenge in intellectual disability research - participants or health and research professionals?

Thank you for the opportunity to review this important and thoughtfully written manuscript. The exclusion of people with intellectual disabilities from clinical research remains a pressing issue, and this study makes a valuable contribution by exploring the recruitment processes that often shape these exclusions. I found the manuscript highly relevant and engaging, and I appreciate the authors' approach to a sensitive and complex topic.

To provide constructive feedback, I would like to highlight several areas where further clarification or elaboration could strengthen the manuscript before publication.

Methods

Topic Guide

The manuscript refers to Supplementary File 1 for details about the topic guide. However, this appears only to include the version currently in use. For transparency and reproducibility, it would also be helpful to include the topic guide used with trial participants with intellectual disabilities.

Participants

Participant characteristics (line 213) are described only briefly. Given the finding that professional experience influenced decisions to include or exclude people with intellectual disabilities, more information about participants' work backgrounds would be valuable.

Additionally, more details about the interviewers would enhance the transparency of the study. For example, what were their backgrounds, what roles did they play in the research process, and how might their perspectives or emotional investment (noted in lines 470 ff.) have shaped data collection or analysis?

It would also be helpful to provide a rationale for the absence of informal caregivers or family members as participants. Their perspectives could have enriched the findings, particularly on the lived experience and practical aspects of trial inclusion.

Data Collection

The manuscript would benefit from more detail about the data collection process:

- Where were the interviews conducted?
- Were participants interviewed alone or accompanied?
- What recruitment strategies were used?
- Was the topic guide piloted or refined during the study?
- Were any interviews repeated?

The discussion of data saturation is limited to a brief mention in the limitations. A more detailed account of how saturation was assessed would strengthen the methodological rigor. In addition, it would be useful to note whether participants were given the opportunity to review their transcripts for accuracy.

Data Analysis

Please clarify whether qualitative analysis software was used.

Additionally, did participants have the opportunity to provide feedback on the findings or interpretations (e.g. through member checking)? If so, that would be worth highlighting.

The process of developing the deductive categories should also be elaborated. A clearer explanation would help readers understand how the analytical framework was constructed.

Discussion

The reference to the UK research framework (line 610 ff) is introduced somewhat abruptly and was not discussed earlier in the paper. It would be helpful to clarify its relevance and implications for the study.

Another useful addition would be a discussion of what kinds of competencies or structural supports researchers may need to engage more meaningfully and consistently with inclusive recruitment practices.

Limitations

Given the size and composition of the sample, it is unlikely that full thematic saturation was achieved, particularly as some stakeholder groups—such as family members—were not included. While the study offers important insights into organisational challenges, this limitation could be more explicitly addressed.

Acknowledging the absence of perspectives from informal caregivers or other relevant stakeholders would further contextualize the findings and their applicability.

In summary, this is a timely and well-conceived study that addresses a significant gap in the literature and practice. You may find the reporting guideline for qualitative research (Consolidated criteria for reporting qualitative research (COREQ): a 32-item checklist for interviews and focus groups - PubMed) helpful for your work. With the mentioned additions and clarifications—particularly in the methods and discussion sections—the manuscript will be well-positioned to make a strong contribution to the field.

Thank you again for the opportunity to review this work.
