# Supplementary material for: Who presents the greatest challenge in intellectual disability research- participants or health and research professionals?
Source: PLoS One. 2025 Nov 3;20(11):e0332744. doi: 10.1371/journal.pone.0332744 (PMC12582445; doi:10.1371/journal.pone.0332744)
Supplement: Attachment — Submitted filename: Response to Reviewers.docx [file pone.0332744.s005.docx]

29/01/2025

Dear editor.

Thank you for your very helpful comments about our manuscript: [PONE-D-24-54259].

Please find below our responses to your comments.

| **Comment** | **Response** |
| --- | --- |
| 1. Please ensure that your manuscript meets PLOS ONE's style requirements, including those for file naming. | Formatting reviewed |
| 2. We note that your Data Availability Statement is currently as follows: [All relevant data are within the manuscript and its Supporting Information files.] Please confirm at this time whether or not your submission contains all raw data required to replicate the results of your study. Authors must share the “minimal data set” for their submission. PLOS defines the minimal data set to consist of the data required to replicate all study findings reported in the article, as well as related metadata and methods. For example, authors should submit the following data:   - The values behind the means, standard deviations and other measures reported;  - The values used to build graphs;  - The points extracted from images for analysis.  Authors do not need to submit their entire data set if only a portion of the data was used in the reported study. If your submission does not contain these data, please either upload them as Supporting Information files or deposit them to a stable, public repository and provide us with the relevant URLs, DOIs, or accession numbers. | We are exercising caution over release of this data even though it is anonymised due to the fact that we are working with a defined population, both in terms of staff and people with ID/T2D. Data, in terms of deidentified interview transcripts, are available from the corresponding author on reasonable request. This is on the condition that the request fulfils the necessary approvals in place for 'controlled access' data, that participants have agreed to the optional consent to share their anonymised data, and that participant anonymity or privacy is considered not to be compromised. |
| 3. Please amend either the title on the online submission form (via Edit Submission) or the title in the manuscript so that they are identical. | Amended |
| 4. Your ethics statement should only appear in the Methods section of your manuscript. If your ethics statement is written in any section besides the Methods, please move it to the Methods section and delete it from any other section. Please ensure that your ethics statement is included in your manuscript, as the ethics statement entered into the online submission form will not be published alongside your manuscript. | The ethics statement is a sub section of the Materials and Methods section in the manuscript |
| **Additional Editor Comments:**   5. This engaging paper iterates inquiries that align well with the reported findings. However, it could be made more precise by explicitly referencing the study's focus on recruitment challenges in randomized controlled trials (RCTs), such as "Identifying the Key Challenges in Intellectual Disability Research Recruitment: Participants or Professionals?" The long title presented is not appropriate. | There was much debate about the title of the manuscript. All the authors came to a consensus that this title was appropriate as it captured the ID/T2D, gatekeeping and QRI elements which we felt all merited equal weight. However, we have reflected on your comments and the authors have agreed to shorten the title and ensure that all of the elements of the paper are captured in the key words to ensure access for as many readers as possible. |
| 6. Employing the QuinteT Recruitment Intervention (QRI) methodology is innovative and well-suited for identifying recruitment barriers and enablers in vulnerable populations. The multi-method approach—interviews, analysis of recruitment discussions, screening logs, and review of study documents—provides a comprehensive view of the challenges. The thematic analysis is appropriately applied, though the lack of screening log data from two sites is a limitation. Including more perspectives from family or carers could have enriched the findings, as their influence on recruitment decisions is significant. | We agree that including more perspectives from participants/family members would have added weight to the results. However, the quotes chosen from the participants were those which were most clearly expressed during the interviews. In addition, we felt it was important to include quotes which would support the main finding- that the bottlenecks lay with the healthcare professionals and systems, so the paper is more weighted to this. Family members were not included in this study, which we have included as a limitation of the study. We now plan to carry out an additional process evaluation with family/paid carers to obtain their views on the benefits/challenges of the study. |
| 7. The conclusions are consistent with the data presented. They highlight the systemic barriers within the healthcare system and among professionals as more significant than participant-related challenges. This finding is insightful and aligns well with the study’s objectives. Focusing on organizational buy-in and emotional labor among healthcare professionals and researchers provides a fresh perspective. However, the conclusions could be more actionable by explicitly connecting the identified challenges to broader implications for healthcare research policy and practice. | We have amended the conclusion section of the manuscript to reflect the current UK Health and Research Policy Framework published by the Health Research Authority in 2023 |
| 8. Citations in the study are appropriate and comprehensive, drawing from a mix of foundational studies and recent advancements. However, some references to key themes, such as advocacy versus paternalism and gatekeeping, could be further developed to deepen the discussion. Additionally, more explicit connections between the study's findings and prior research on RCT challenges in vulnerable populations would strengthen the narrative. | We have added the following reference to reflect prior research into the issues relating to gatekeeping and engagement:  *Earle et al 2020 A critical reflection on accessing women with learning disabilities to participate in research about sensitive subjects through organisaiotnal partnerships. Br J Learn Disabil 48:162-169* |
| 9. Ensure a balanced discussion of participant versus professional challenges by integrating more voices from participants or caregivers where possible. | See response to point 6 |
| 10. Tighten the narrative in the discussion section to link findings to actionable recommendations and broader implications. | We have added an introductory sentence to the discussion section in relation to real-world applicability. We feel the rest of the discussion section does link to our amended conclusions and stays true to the experiences of everyone involved in the study. |
| 11. Incorporating recent literature can enhance your study by providing contemporary insights into recruitment challenges in intellectual disability (ID) research and related fields. Here are some pertinent studies:   (a). Shariq, S., Cardoso Pinto, A.M., Budhathoki, S.S. et al. Barriers and facilitators to the recruitment of disabled people to clinical trials: a scoping review. Trials 24, 171 (2023).  (b). Iflaifel, M., Hall, C.L., Green, H.R. et al. Widening participation – recruitment methods in mental health randomised controlled trials: a qualitative study. BMC Med Res Methodol 23, 211 (2023).  ©. McCausland D, Haigh M, McCallion P, and McCarron M. IRB challenges in multisite studies: A case report and commentary from the Intellectual Disability Supplement to the Irish Longitudinal Study on Ageing (IDS-TILDA) [version 1; peer review: 3 approved]. HRB Open Res 2024, 7:3  (d). Jacobsen P, Haddock G, Raphael J, Peak C, Winter R, Berry K. Recruiting and retaining participants in three randomised controlled trials of psychological interventions conducted on acute psychiatric wards: top ten tips for success. BJPsych Open. 2022;8(4):e125. doi:10.1192/bjo.2022.527. Summary: Although focused on psychiatric settings, this article provides practical recruitment and retention strategies that could be adapted for ID research contexts.  €. Jones H, Cipriani A, Barriers and incentives to recruitment in mental health clinical trials. BMJ Ment Health 2019;22:49-50. Summary: This article examines factors influencing recruitment in mental health trials, offering insights that may be relevant to overcoming similar challenges in ID research. | Thank you for these helpful references. We have included the reference from Shariq as it refers to people with ID. The other references while very interesting focus more on those with mental health issues which is not the remit of this paper. |
| 12. Incorporate the one area missing in this manuscript, the recent moves by regulatory agencies towards Real World Evidence (RWE) trials that will be supported by the conclusions drawn in this paper, with discussions on recruitment challenges in clinical research involving vulnerable populations. The FDA’s 2023 Guidance on Diversity Plans to Improve Enrollment of Participants from Underrepresented Racial and Ethnic Populations in Clinical Trials offers insights on planning and implementing inclusive recruitment strategies. While this guidance is not ID-specific, its principles could be adapted for this study's context. | We do not feel it is appropriate to use FDA guidance in a manuscript describing a study carried out in the UK. However, your suggestions were helpful in identifying some additional information for inclusion relating to UK guidance. To this end we have included some detail on the guidance contained within the HRA Research in Healthcare Framework and the National Institute for Health and Care Excellence (NICE) real-world evidence framework, which apply across the 4 nations of the UK and therefore directly apply to this study. |
| 13. Integrate the FDA's emphasis on investigator training for inclusive recruitment and apply it to the context of ID studies. | As above |
| 14. Reference recent literature on recruitment challenges in vulnerable populations (such as the 2023 scoping review on disabled populations) to position the study within the broader clinical research landscape. | See point 11 |
| 15. Include a recommendation to adopt structured recruitment plans that align with FDA diversity guidelines. This could involve pre-trial simulations or mock recruitment to identify bottlenecks early. | See point 12 |
| 16. Conclude by advocating for integrating regulatory frameworks, such as FDA guidance, into future ID research to standardize recruitment practices and improve inclusivity. | See point 12 |
| 17. Discuss the FDA's 2023 Guidance on Diversity Plans in the methods and recommendations sections to underscore the importance of structured recruitment strategies. | See point 12 |
| 18. Cite the FDA’s Guidance for Industry on Clinical Trial Design and Conduct for Pediatric Populations for parallels in designing trials for vulnerable populations. | See point 12 |
| 19. Highlight how the FDA’s focus on inclusivity can complement ethical frameworks like the Declaration of Helsinki in the study's context. | See point 12 |

Yours Sincerely,

Dr Rosemary Kelly
