# Supplementary material for: Who presents the greatest challenge in intellectual disability research- participants or health and research professionals?
Source: PLoS One. 2025 Nov 3;20(11):e0332744. doi: 10.1371/journal.pone.0332744 (PMC12582445; doi:10.1371/journal.pone.0332744)
Supplement: Attachment — Submitted filename: Response to Reviewers 06032025.docx [file pone.0332744.s006.docx]

06/03/2025

Dear editor.

Thank you for pointing out the remaining errors in our manuscript: [PONE-D-24-54259].

Please find below confirmation of the required amendments:

| **Ref No** | **Reference** | **Amendment** |
| --- | --- | --- |
| 9. | van Schrojenstein HM, Lantman-de Valk J, Noonan Walsh P. Managing Health Problems in People with Intellectual Disabilities. BMJ. 2008; 337(7683): 1408-1412. doi: 10.1136/bmj.a2507 | Additional wording added - (also see Correction: BMJ 2009;339 (771): 42). |
| 12. | Bishop R, Laugharne R, Shaw N, Russell AM, Goodley D, Banerjee S, et al. The inclusion of adults with intellectual disabilities in health research – challenges, barriers and opportunities: a mixed-method study among stakeholders in England. J Intell Disabil Res. 2024;68(2): 140-149. doi: 10.1111/jir.13097 | Date of publication amended |
| 31. | Taggart L, Truesdale M, Carey ME, Martin-Stacey L, Scott J, Bunting B, et al. Pilot feasibility study examining an structured self-management diabetes education program, DESMOND-ID, targeting Hb1Ac in adults with intellectual disabilities. Diabetes Med. 2018;35(1): 137-146. doi: 10.1111/dme.13539 | Date of publication amended |
| 47. | Corby D, Sweeney MR. Researchers’ experiences and lessons learned from doing mixed-methods research with a population with intellectual disabilities: Insights from the SOPHIE study. J Intell Disabil. 2019;23(2): 250-265. doi: 10.1177/1744629517747834 | Date of publication amended |
| 53. | Meadmore K, Church H, Crane K, Blatch-Jones A, Recio Saucdo AR, Fackrell K. An in-depth exploration of researcher experiences of time and effort involved in health and social care research funding in the UK: The need for changes. PLoS ONE. 2023;18(9): e0291663.  doi: 10.1371/journal.pone.0291663 | Date of publication amended |

Yours Sincerely,

Dr Rosemary Kelly
