# Supplementary material for: Who presents the greatest challenge in intellectual disability research- participants or health and research professionals?
Source: PLoS One. 2025 Nov 3;20(11):e0332744. doi: 10.1371/journal.pone.0332744 (PMC12582445; doi:10.1371/journal.pone.0332744)
Supplement: Attachment — Submitted filename: Response to reviewers2.docx [file pone.0332744.s008.docx]

Response to reviewers

| **Comment** | **Response** |
| --- | --- |
| **Topic Guide**  The manuscript refers to Supplementary File 1 for details about the topic guide. However, this appears only to include the version currently in use. For transparency and reproducibility, it would also be helpful to include the topic guide used with trial participants with intellectual disabilities. | The topic guide for participants with ID has been added as Supplementary File 3 |
| **Participants**  Participant characteristics (line 213) are described only briefly. Given the finding that professional experience influenced decisions to include or exclude people with intellectual disabilities, more information about participants’ work backgrounds would be valuable. | This sample was derived from a group of professionals within a specific field of practice in the United Kingdom. The authors are limited in any additional details they can include to avoid risking the participants’ anonymity which was assured as part of the consent process. We have included some general information to illustrate the diversity of roles. |
| More details about the interviewers would enhance the transparency of the study. For example, what were their backgrounds, what roles did they play in the research process, and how might their perspectives or emotional investment (noted in lines 470 ff.) have shaped data collection or analysis? | We have included a short descriptor of author 1 who was the interviewer throughout this study. |
| It would also be helpful to provide a rationale for the absence of informal caregivers or family members as participants. Their perspectives could have enriched the findings, particularly on the lived experience and practical aspects of trial inclusion. | We have recognised this as a limitation of the study and with hindsight should have included interviews with carers. We hope to address this with a further piece of work to capture their views and have stated this in the Strengths and Limitations Section |
| **Data Collection**  The manuscript would benefit from more detail about the data collection process:   - Where were the interviews conducted? - Were participants interviewed alone or accompanied? - What recruitment strategies were used? - Was the topic guide piloted or refined during the study? - Were any interviews repeated? | Some of this information is available in the COREQ table and we have also added some further detail about the interviews in the main manuscript following Table 1 |
| The discussion of data saturation is limited to a brief mention in the limitations. A more detailed account of how saturation was assessed would strengthen the methodological rigor. In addition, it would be useful to note whether participants were given the opportunity to review their transcripts for accuracy. | We were not seeking data saturation in this study. We sought to explore the views of people engaged in the internal pilot of the RCT to identify potential enablers and barriers to recruitment and were keen to collect a variety of opinions and experiences to enhance recruitment to the main trial.  Participants were not asked to review their transcripts. A meeting which included many of the professionals interviewed reviewed an early version of the data analysis and provided further information and clarity. The individuals with intellectual disabilities were not asked to review their transcripts as this was felt to be an additional burden for them, but a lay summary of the results has been provided in an easy-read format. This is alluded to on page 23 but following your advice, we have now added a short paragraph on p10 to clarify this. |
| **Data Analysis**   - Please clarify whether qualitative analysis software was used. - Additionally, did participants have the opportunity to provide feedback on the findings or interpretations (e.g. through member checking)? If so, that would be worth highlighting. - The process of developing the deductive categories should also be elaborated. A clearer explanation would help readers understand how the analytical framework was constructed. | - No software was used. Interviews were transcribed and coded as described in the main manuscript. - Member checking with professionals took place during an investigator meeting where several of those who took part in the interviews were present. - A further graphic has been included to illustrate how the categories were developed (Fig1) |
| **Discussion**   - The reference to the UK research framework (line 610 ff) is introduced somewhat abruptly and was not discussed earlier in the paper. It would be helpful to clarify its relevance and implications for the study. - Another useful addition would be a discussion of what kinds of competencies or structural supports researchers may need to engage more meaningfully and consistently with inclusive recruitment practices. | - The UK framework was added following advice in an earlier review. However, we accept that we should have explored this earlier in the manuscript and have now moved this to the Introduction. - We have added some further thoughts about potential competencies and support for researchers involved in recruitment of vulnerable populations: - Identifying how/where potential participants can be approached - community groups, day centres etc - Communication skills about explaining research studies - Understanding selection/gatekeeper bias - Research processes |
| **Limitations**  Given the size and composition of the sample, it is unlikely that full thematic saturation was achieved, particularly as some stakeholder groups—such as family members—were not included. While the study offers important insights into organisational challenges, this limitation could be more explicitly addressed.  Acknowledging the absence of perspectives from informal caregivers or other relevant stakeholders would further contextualize the findings and their applicability. | Braun & Clark, whose thematic analysis framework was used in this work advise avoiding claims of saturation, preferring the use of concepts such as ‘information power’. We feel our thematic analysis, in identifying the multiplicity of organisational challenges which can provide learning not just for researchers in the intellectual disability field but for all researchers is an example of how we have met the aims and objectives of this QRI study.  As previously stated, we have recognised the absence of data from carers as a limitation of the study. We hope to address this with a further piece of work to capture their views and have stated this in the Strengths and Limitations Section |
